# Endoscopic ultrasonography evaluation combined with guidewire-guided cystotome therapy for refractory benign esophageal stricture: Improving the safety of treatment

**DOI:** 10.1055/a-2523-2821

**Published:** 2025-02-11

**Authors:** Chen Shi, Chen Chen Dai, Qiao Mei

**Affiliations:** 1 36639 Department of Gastroenterology, The First Affiliated Hospital of Anhui Medical University, Hefei, China

Here we report a patient with a refractory benign esophageal stricture treated using endoscopic ultrasound (EUS) evaluation combined with guidewire-guided cystotome therapy as a new endoscopic approach.

The gastroscopy of a 32-year-old patient with dysphagia after two endoscopic dilatations revealed complete esophageal stricture about 30 cm from the incisors ( Fig. 1 **a** ). Preoperative small-probe EUS showed that the most severe stricture of the wall thickened to 1.0 cm with a length of 3 cm, and no obvious structural damage was observed ( Fig. 1 **b** ). A yellow Zebra guidewire (Boston Scientific, Marlborough, Massachusetts, USA) was first passed through the stricture, followed by a 6-Fr cystotome with a diathermic metal tip (Cysto Gastro-Set; ENDO-FLEX, Voerde, Germany) through guidewire to the stricture. Current was applied until the tip of the cystotome crossed the stricture and severed the muscle layer ( Fig. 2 **a** ) using a standard sphincterotomy setting and maintaining Endo-Cut I mode (monopolar, forced coagulation, Effect-3, Coag 40 watts) ( Video 1 ). EUS was used repeatedly to assess the thickness of the esophageal wall to guide the incision direction throughout the process ( Fig. 2 **b** ). Finally, a lumen diameter of 1.2 cm was achieved, enabling the gastroscope to pass through ( Fig. 3 **a, b** ). The patient responded well without major complications.

**Fig. 1:**
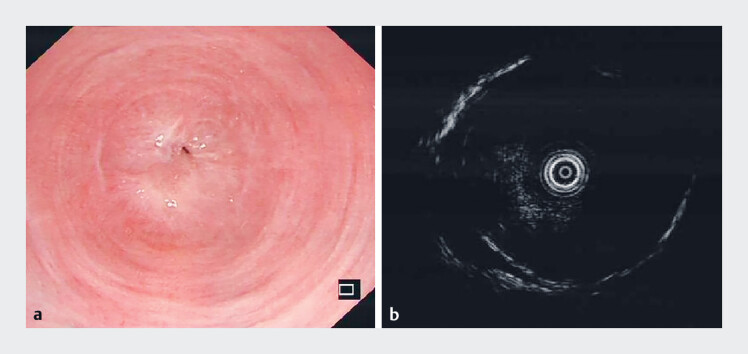
Complete esophageal stricture. **a** Gastroscopy image of complete esophageal stricture about 30 cm from the incisors. **b** The most severe stricture of the esophageal wall thickened to 1.0 cm with a length of 3 cm, and no obvious structural damage was observed in the small-probe endoscopic ultrasound (EUS) image.

**Fig. 2:**
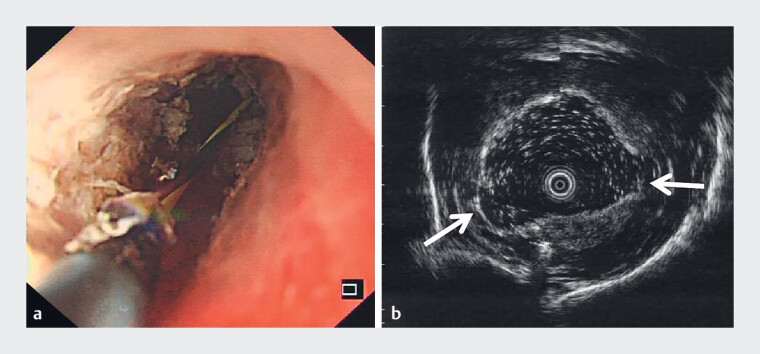
Incision therapy with EUS evaluation combined with guidewire-guided cystotome. **a** A 6-Fr cystotome with a diathermic metal tip crossed through the yellow Zebra guidewire to the stricture, and current was applied up to the tip to sever the muscle layer. **b** EUS image of the muscle layer being severed during incision (indicated by arrows).

**Fig. 3:**
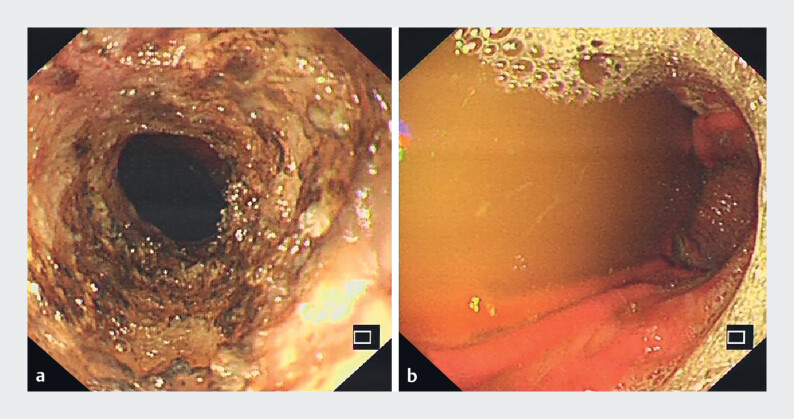
Gastroscopic images after incision treatment. **a** Gastroscopic image after complete incision of esophageal stricture. **b** Gastroscope could reach the stomach through the stricture.

Endoscopic ultrasound evaluation combined with guidewire-guided cystotome therapy for refractory benign esophageal stricture.Video 1

Dilation with bougies or balloons is the classic endoscopic treatment for benign esophageal strictures 1 , but over 30% of patients need continuing dilation for more than two sessions during long-term follow-up 2 . Martínez-Guillén et al. described the management of complete gastrointestinal strictures using an EUS-guided puncture, but one of the four cases failed due to a long stricture (>3 cm) 3 . In our case, the use of small-probe EUS to repeatedly evaluate the thickness of the stricture and the depth of incision allowed for more precise treatment, and using a cystotome to pass through the guidewire allowed accurate control of the direction of the incision, thereby reducing the incidence of perforation complications.

This therapy could be a new method and an extremely safe and useful accessory in refractory benign esophageal strictures.

Endoscopy_UCTN_Code_TTT_1AO_2AH

## Notes

Endoscopy E-Videos https://eref.thieme.de/e-videos
*E-Videos* is an open access online section of the journal *Endoscopy* , reporting on interesting cases and new techniques in gastroenterological endoscopy. All papers include a high-quality video and are published with a Creative Commons CC-BY license. Endoscopy E-Videos qualify for HINARI discounts and waivers and eligibility is automatically checked during the submission process. We grant 100% waivers to articles whose corresponding authors are based in Group A countries and 50% waivers to those who are based in Group B countries as classified by Research4Life (see: https://www.research4life.org/access/eligibility/ ). This section has its own submission website at https://mc.manuscriptcentral.com/e-videos .

## References

[1] Standards of Practice Committee Egan JV Baron TH Esophageal dilation Gastrointest Endosc 2006 63 755 760 16650533 10.1016/j.gie.2006.02.031

[2] de Wijkerslooth LR Vleggaar FP Siersema PD Endoscopic management of difficult or recurrent esophageal strictures Am J Gastroenterol 2011 106 2080 2092 10.1038/ajg.2011.348 22008891

[3] Martínez-Guillén M Gornals JB Consiglieri CF EUS-guided recanalization of complete gastrointestinal strictures Rev Esp Enferm Dig 2017 109 643 647 10.17235/reed.2017.4972/2017 28724308

